# Two-dimensional speckle tracking echocardiography in chemotherapy-induced cardiotoxicity in females with breast cancer

**DOI:** 10.1186/s40959-024-00209-1

**Published:** 2024-03-01

**Authors:** Ahmed A. Fawzy, Khaled A. El-Menyawi, Walid M. Sallam, Mohamed E. Zahran

**Affiliations:** Department of Cardiology, Faculty of Medicine, Ain Shams University, Cairo, Egypt

## Abstract

**Background:**

Cancer and cardiovascular diseases are the main causes of mortality worldwide. Although the incidence of cancer is rising, modern comprehensive management including surgery, chemotherapy, and radiotherapy led to decreased mortality, but also different cardiovascular complications. Conventional EF measurement fails to detect subtle changes in LV function, so a more sensitive tool is needed.

**Methods:**

The study included 101 asymptomatic female patients with newly diagnosed breast cancer who received anthracycline ± trastuzumab-based chemotherapy regimen. A comprehensive echocardiographic examination was performed before receiving the chemotherapy (T0), at 3 months (T1), and at 6 months after (T2). All patients had pre-treatment normal LV EF. Asymptomatic CTRCD is defined as: severe if new LVEF reduction to < 40%, moderate if new LVEF reduction by ≥ 10 percentage points to an LVEF of 40–49% or new LVEF reduction by, 10 percentage points to an LVEF of 40– 49% and either new relative decline in GLS by .15% from baseline or new rise in cardiac biomarkers and mild if LVEF ≥ 50% and new relative decline in GLS by .15% from baseline and/or new rise in cardiac biomarkers. Symptomatic CTRCD is defined as: very severe if HF requiring inotropic support, mechanical circulatory support, or consideration of transplantation, severe if required hospitalization, moderate if required outpatient intensification of diuretic and HF therapy and mild if there are mild HF symptoms and no intensification of therapy required according to the latest ESC cardio oncology guidelines. The Lower reference value set for RV S’ was less than 10cm/s to define RV systolic dysfunction according to ASE guidelines.

**Results:**

CTRCD occurred in 24 patients (25.5%) while RV systolic dysfunction was more common occurring in 37 patients (39.4%). LV GLS at (T1) (cut-off value < -15% with relative 12.5% reduction from the baseline value) was a strong predictor of CTRCD, but combining LV GLS with RV GLS & RV FWLS was the strongest (AUC = 0.947, sensitivity = 91.67%, specificity = 90%).

**Conclusion:**

Chemotherapy induces biventricular changes with more prevalent deterioration in RV values. Low LV & RV strain values at baseline together with reduction of these values after chemotherapy treatment can predict later CTRCD development. Combining LV GLS with RV GLS & FWLS values at (T1) is the strongest predictor of subsequent CTRCD.

## Introduction

Breast cancer is the most common cancer among females worldwide, and it is ranked as the fifth leading cause of cancer death [1]. Anthracyclines and HER-2 inhibitor (Trastuzumab) are the mainstays of chemotherapy for breast cancer and although they prolonged survival in patients, they led to cardiac toxicity which ranges from mild asymptomatic decrease in EF to clinically overt form of heart failure.

Anthracyclines lead to cardiotoxicity in a dose-dependent manner with a 5% incidence of heart failure with an average cumulative dose of 400 mg/m^2^, but it can reach up to 48% with an average cumulative dose of 700 mg/m^2^ [2]. Conversely, Trastuzumab-induced cardiotoxicity is not dose-dependent and is thought to be due to changing the expression of genes responsible for DNA repair and mitochondrial function leading to impaired systolic function [3].

Traditional follow-up of chemotherapy patients was done by LV EF measurement together with clinical surveillance of heart failure symptoms. Despite the prognostic value of EF measurement, it lacks sensitivity to detect early changes in myocardial function [4]*.* This is due to many pitfalls including for example inadequate LV apex visualization, the need for geometrical assumption for calculations, the variability of measurements [5] and also being dependent on loading conditions, etc.…

Because of the aforementioned causes, LVEF reduction is often detected late with failure to recover in 58% of the cases [6]. Hence, there is a growing interest in identifying the earliest markers of myocardial impairment that can predict subsequent drops in EF. LV strain parameters measurement was used for early assessment of myocardial changes [7] and LV GLS was adopted by ASE/EACVI expert consensus to be used for follow-up during the chemotherapy treatment [8].

There is a lack of literature about the effect of chemotherapy on RV function and strain parameters, whether LV changes precede RV changes or the opposite. Also, it’s questionable if changes in GLS values for the RV could predict LV EF drop or not. In this study, we aimed to study changes in LV & RV mechanics during the chemotherapy treatment and detect the strongest predictor of subsequent cardiotoxicity.

## Methodology

This is an observational prospective study that included 101 asymptomatic female patients who were scheduled to receive chemotherapy for breast cancer during the period from April 2021 to December 2022.

Exclusion criteria included history of previous chemotherapy or radiotherapy, reduced LV EF < 50%, a history of coronary artery disease, patients with severe valvular disease or primary cardiomyopathy, history of medical diseases affecting right ventricular function, and patients with Atrial Fibrillation or with permanent pacemakers.

All patients received anthracycline (doxorubicin or epirubicin) in 3-week cycles for an average of 3–4 cycles. HER-2 (Human Epidermal growth factor Receptor-2) inhibitor (Trastuzumab) was administered in some patients and none of the study population received concurrent anthracyclines and Trastuzumab. Traditional cardiovascular risk factors such as hypertension, diabetes, chronic kidney disease, smoking, hypothyroidism, and obesity (BMI) were recorded in our study population.

All patients were informed about the study and informed consent was obtained. This study was approved by Ain-shams University's ethical committee & complied with the ethical guidelines of the 1975 declaration of Helsinki as revised in 2008.

### The baseline visits

All patients underwent thorough history-taking regarding demographic data, previous oncological therapy, and cardiovascular risk factors. Hypertension was defined as patients taking antihypertensive treatment or patients with Systolic BP ≥ 140 mmHg or Diastolic BP ≥ 90 mmHg based on the mean of two or more properly measured seated blood pressure readings on two or more occasions [9]. Patients with HbA1C ≥ 6.5% or at least two fasting glucose levels ≥ 126 mg/dL or in use of oral hypoglycemic drugs and/or insulin were classified as diabetics [10]. Chronic kidney disease was defined as kidney damage or glomerular filtration rate < 60 ml/min/1.73 m2 for 3 months or more [11]. Data regarding the chemotherapy regimen and received doses were obtained from patients’ files.

### Two dimensional transthoracic Echocardiography (TTE)

Resting ECG-gated transthoracic echocardiography was performed for all patients. Patients were imaged in the left lateral decubitus position using a General Electric (GE) Vingmed Ultrasound Vivid 5 or Vivid 7 system. Full 2D, M-mode, and Doppler images in standard precordial apical and parasternal views were stored and digitally recorded for subsequent analysis. Each measurement was taken from the average of 3 continuous cardiac cycles. Left atrial (LA) diameter, LV end-systolic dimension (LVESD), LV end-diastolic dimension (LVEDD), and LV wall thickness were measured in the parasternal long-axis view. LV diastolic function was assessed by Mitral inflow pattern (E/A ratio), E velocity deceleration time, annular tissue Doppler curves (e’/a’), and E/e’ ratio. LVEF was calculated using modified Simpson’s method. Asymptomatic CTRCD is defined as: severe if new LVEF reduction to < 40%, moderate if new LVEF reduction by ≥ 10 percentage points to an LVEF of 40–49% or new LVEF reduction by, 10 percentage points to an LVEF of 40– 49% and either new relative decline in GLS by 0.15% from baseline or new rise in cardiac biomarkers and mild if LVEF ≥ 50% and new relative decline in GLS by 0.15% from baseline and/or new rise in cardiac biomarkers. Symptomatic CTRCD is defined as: very severe if HF requiring inotropic support, mechanical circulatory support, or consideration of transplantation, severe if required hospitalization, moderate if required outpatient intensification of diuretic and HF therapy and mild if there are mild HF symptoms and no intensification of therapy required according to the latest ESC cardio oncology guidelines [12]. RV systolic function was assessed using systolic TV annular excursion velocity (S’). The lower reference value of S’ was set as 10 cm/s. All the measurements were performed and reported according to the recommendations of the American Society of Echocardiography [13].

### Measurement of myocardial strain

The standard three apical views together with the parasternal short axis view at the level of insertion of the papillary muscles were used to obtain LV strain values (LV longitudinal (GLS), circumferential (GCS), and radial (GRS)) and the focused RV apical four-chamber view for global longitudinal peak RV systolic strain (RV GLS) and longitudinal peak systolic strain of the RV free wall (RV FWLS) [14].

Three consecutive heart cycles were recorded and averaged. The frame rate was set between 60 and 80 frames per second; these settings were recommended to combine temporal resolution with adequate spatial definition and to enhance the feasibility of the frame-to-frame tracking technique. Offline analysis was performed using the commercially available software (EchoPAC; GE Medical Systems). Segments of the LV with inadequate image quality were excluded from the analysis due to the presence of acoustic shadows, image artifacts, reverberations, or poor quality in the trace of the points by manual evaluation.

The RV peak systolic global longitudinal strain (RVGLS) was measured from all 6 RV myocardial segments from an apical 4-chamber view (3 segments of the free wall plus 3 segments of the interventricular septum) while the RV free wall peak systolic longitudinal strain (RVFWLS) was obtained from the 3 RV free wall segments only. Both were measured because in RVGLS the interventricular septum is measured taking into consideration that it might partially reflect changes in the left ventricle as the septum is shared by both ventricles. While measuring RVFWLS focus is only on the RV-free wall and does not include the contribution of the septum; however, it doesn’t consider potential changes that may occur in the RV septum.

### The follow-up visits

Patients were asked to follow up after 3 and 6 months from the start of the chemotherapy. In every follow-up visit, revision of the chemotherapy regimen and proper clinical assessment for symptoms and signs of heart failure were done. Echocardiographic imaging (conventional and 2D-STE) was repeated, and the same study parameters were recorded. Patients in the 3-month follow-up visits who met the definition of CTRCD received the proper anti-failure treatment according to the latest ESC guidelines in 2021.

### Statistical analysis

All data were plotted into tables and statistically studied. All statistical analyses were performed with SPSS software (version 12.0, SPSS Inc., Chicago, Illinois). Quantitative variables were expressed as mean ± standard deviation and compared by Student’s *t-test*, whereas qualitative variables were expressed by their frequencies and percentages, and compared by the chi-square test. A *p*-value < 0.05 was considered statistically significant. Univariate and multivariate linear regression analysis were used with demographic and echocardiographic data introduced into the regression model to identify the independent predictors of cardiotoxicity. A receiver operator characteristic (ROC) analysis was used to detect the best cut-off values in strain values with the highest sensitivity and specificity for the prediction of future cardiotoxicity.

## Results

In the current study, 101 female patients diagnosed with non-metastatic breast cancer were initially recruited. Six patients were lost to follow-up, and 1 patient died before the first follow-up visit after 3 months, so finally we have 94 patients included in the statistical analysis at the follow-up visits.

### Patient characteristics and chemotherapy regimens

The baseline demographic and clinical data of the study population are summarized in Table 1. The mean age of the study population was 48.84 ± 10.17 years old. None of the patients was an active smoker. The mean BSA of the study population was 1.83 ± 0.19 kg/m2.

**Table 1:** Demographic data and characteristics of the studied patients

		**Total no. = 94**
Gender	Female	94 (100.0%)
Age	Mean ± SD	48.84 ± 10.17
	Range	20 – 70
BSA	Mean ± SD	1.83 ± 0.19
	Range	1.5 – 2.3
Diabetes Mellitus	No	62 (66.0%)
	Yes	32 (34.0%)
Hypertension	No	67 (71.3%)
	Yes	27 (28.7%)
Chronic Kidney Disease	No	93 (98.9%)
	Yes	1 (1.1%)
Thyroid Disorders	Normal	90 (95.7%)
	Hypothyroid	4 (4.3%)

All patients received an anthracycline-based chemotherapy regimen (doxorubicin “Adriamycin” or epirubicin) in addition to cyclophosphamide. Forty-seven patients received Trastuzumab as shown in Table 2.

**Table 2:** Chemotherapy regimen details among the studied patients

		**Total no. = 94**
Chemotherapy Regimen	AC	43 (45.7%)
	EC	51 (54.3%)
Trastazumab	No	47 (50.0%)
	Yes	47 (50.0%)
Cumulative Dose of Doxorubicin (mg)	Mean ± SD	435.26 ± 45.6
	Range	370 – 552
Cumulative Dose of Epirubicin (mg)	Mean ± SD	731.76 ± 75.5
	Range	600 – 896

When indexing these values to BSA (the mean cumulative dose of doxorubicin is 239 mg/m2 and of epirubicin is 400 mg/m2).

### Incidence of Cancer Therapy Related Cardiac Dysfunction (CTRCD), right ventricular dysfunction

At the end of the follow-up period, CTRCD occurred in 24 patients, 11 patients (45.8%) after 3 months (T1) and 13 (54.2%) after 6 months (T2). In these patients, none showed cardiac-related symptoms. Interestingly, Right ventricular dysfunction (defined as RV S’ < 10 cm/s) was more common occurring in 37 patients representing 39.4% of the study population. 18 patients had Right ventricular dysfunction at T1 and 19 patients at T2.

### CTRCD vs non-CTRCD cases at baseline echocardiography

The baseline parameters present in Table 3 showed that CTRCD cases had lower mean EF at baseline, but this was not statistically significant when assessed by Simpson’s method. The mean LV GLS was -18.85 ± 1.56% in non-CTRCD cases and -17.58 ± 1.28% in CTRCD cases which was statistically significant. LV GCS was also statistically different while LV GRS didn’t achieve statistical significance. RV S’, RV GLS, and RV FWLS were lower in CTRCD cases and this was statistically significant.

**Table 3:** Comparison between cases with and without CTRCD regarding ECHO parameters at baseline

Baseline		Without CTRCD	With CTRCD	Test value	*P*-value	Sig
		**No. = 70**	**No. = 24**			
LVEDD	Mean ± SD	47.14 ± 4.12	48.42 ± 3.59	-1.348^b^	0.181	NS
	Range	39 – 55	44 – 56			
LVESD	Mean ± SD	30.73 ± 3.25	32.67 ± 3.23	-2.527^b^	0.013	S
	Range	23 – 37	28 – 40			
LA AP diameter	Mean ± SD	35.59 ± 4.11	35.96 ± 2.77	-0.412^b^	0.681	NS
	Range	26 – 45	30 – 42			
Diastolic function	Normal	16 (22.9%)	1 (4.2%)	4.214^a^	0.040	S
	Grade I	54 (77.1%)	23 (95.8%)			
	Grade II	0 (0.0%)	0 (0.0%)			
	Grade III	0 (0.0%)	0 (0.0%)			
EF by M-mode	Mean ± SD	63.67 ± 3.94	61.29 ± 3.28	2.660^b^	0.009	HS
	Range	58 – 74	54 – 68			
EF by Simpson’s method	Mean ± SD	60.21 ± 2.80	59.29 ± 3.50	1.304^b^	0.195	NS
	Range	52 – 68	53 – 68			
RV S’	Mean ± SD	12.89 ± 0.86	12.42 ± 0.83	2.325^b^	0.022	S
	Range	11 – 15	11 – 14			
GLS %	Mean ± SD	-18.85 ± 1.56	-17.58 ± 1.28	-3.603^b^	0.001	HS
	Range	-22 – -16	-21 – -16			
GCS %	Mean ± SD	-24.34 ± 1.77	-23.21 ± 1.86	-2.647^b^	0.010	S
	Range	-27.6 – -21	-26.6 – -20.9			
GRS %	Mean ± SD	45.91 ± 6.55	43.76 ± 6.83	1.370^b^	0.174	NS
	Range	33.8 – 58.8	37.2 – 55.2			
RVGLS %	Mean ± SD	-23.20 ± 1.04	-22.68 ± 0.92	-2.170^b^	0.033	S
	Range	-27 – -21.6	-25 – -21.6			
RV FWLS %	Mean ± SD	-25.47 ± 1.20	-24.83 ± 0.96	-2.354^b^	0.021	S
	Range	-30 – -23	-27 – -23			

^a^Chi-square test

^b^Independent t-test

### CTRCD vs non-CTRCD cases at 3-month follow-up echocardiography

Table 4 shows the difference regarding baseline echocardiography parameters between CTRCD cases and non-CTRCD cases. LV EF, LV GLS, and LV GCS were lower in CTRCD cases in a statistically significant value. LV GRS was also lower in CTRCD cases, but not statistically significant. RV S’, RV GLS & RV FWLS were lower with a statistical significance in CTRCD cases.

**Table 4:** Comparison between cases with and without CTRCD regarding ECHO parameters after 3 months

After 3 months		Without CTRCD	With CTRCD	Test value	*P*-value	Sig
		**No. = 70**	**No. = 24**			
LVEDD	Mean ± SD	47.84 ± 3.87	50.88 ± 4.50	-3.173^b^	0.002	HS
	Range	40 – 56	43 – 58			
LVESD	Mean ± SD	31.79 ± 3.43	37.42 ± 4.36	-6.464^b^	0.000	HS
	Range	25 – 39	29 – 46			
LA	Mean ± SD	36.53 ± 3.45	37.83 ± 2.87	-1.664^b^	0.099	NS
	Range	28 – 45	32 – 45			
Diastolic degree	Normal	11 (15.7%)	1 (4.2%)	2.140^a^	0.143	NS
	Grade I	59 (84.3%)	23 (95.8%)			
	Grade II	0 (0.0%)	0 (0.0%)			
	Grade III	0 (0.0%)	0 (0.0%)			
EF M mode	Mean ± SD	62.07 ± 4.06	52.50 ± 5.65	8.974^b^	0.000	HS
	Range	55 – 74	35 – 60			
EF Simpson’s method	Mean ± SD	59.13 ± 2.53	49.54 ± 5.63	11.356^b^	0.000	HS
	Range	54 – 65	35 – 59			
RV S’	Mean ± SD	12.74 ± 1.29	9.42 ± 0.97	11.515^b^	0.000	HS
	Range	9 – 15	8 – 11			
GLS %	Mean ± SD	-17.80 ± 1.88	-13.54 ± 1.91	-9.539^b^	0.000	HS
	Range	-22 – -13	-19 – -9			
GCS %	Mean ± SD	-24.37 ± 1.73	-21.83 ± 2.51	-5.488^b^	0.000	HS
	Range	-28 – -21	-26 – -15			
GRS %	Mean ± SD	45.00 ± 6.28	42.08 ± 8.03	1.825^b^	0.071	NS
	Range	32 – 58	26 – 57			
RV GLS%	Mean ± SD	-22.14 ± 2.92	-15.38 ± 2.57	-10.101^b^	0.000	HS
	Range	-26 – -13	-24 – -12			
RV FWLS %	Mean ± SD	-24.36 ± 3.18	-17.13 ± 2.42	-10.163^b^	0.000	HS
	Range	-28 – -15	-26 – -14			

^a^Chi-square test

^b^Independent t-test

### Changes in LV and RV echocardiographic parameters at T0, T1 and T2

**Fig. 1:**
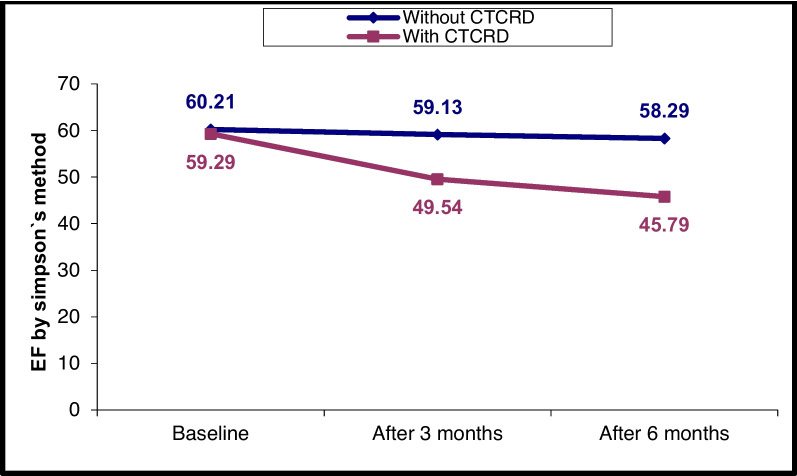
EF by Simpson’s method changes in CTRCD and non-CTRCD cases at baseline, after 3 months and after 6 months

**Fig. 2:**
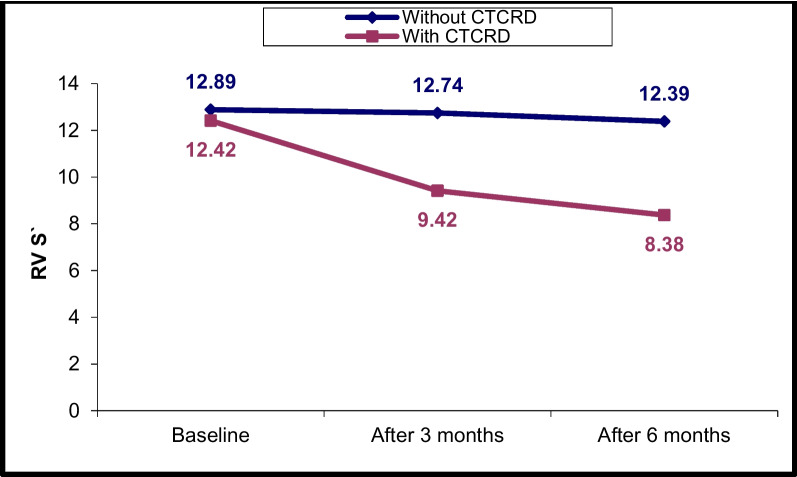
RV S’ changes in CTRCD and non-CTRCD cases at baseline, after 3 months and after 6 months

**Fig. 3:**
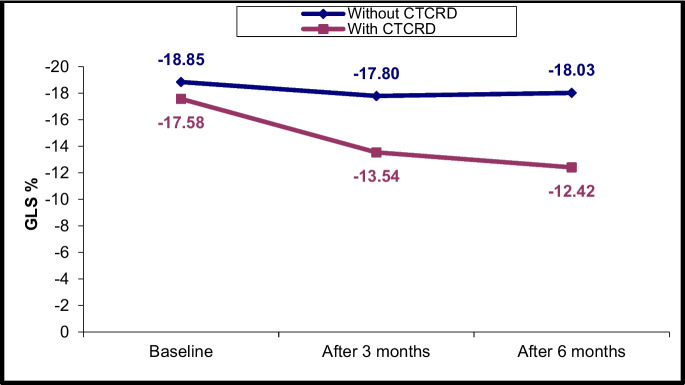
LV GLS% changes in CTRCD and non-CTRCD cases at baseline, after 3 months and after 6 months

**Fig. 4:**
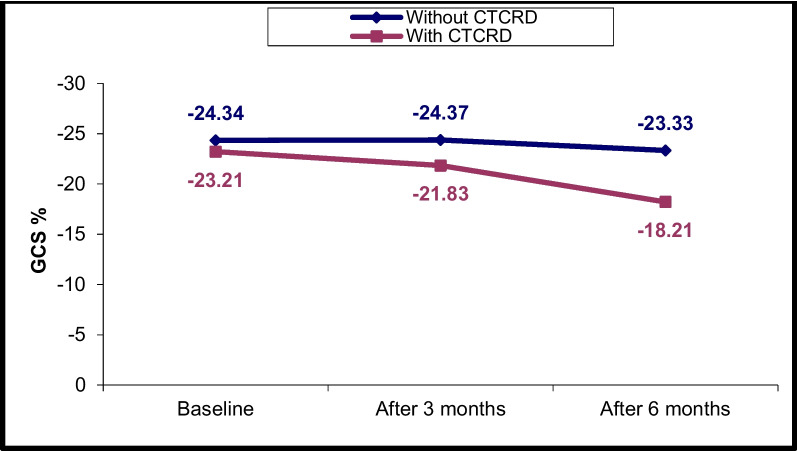
LV GCS% changes in CTRCD and non-CTRCD cases at baseline, after 3 months and after 6 months

**Fig. 5:**
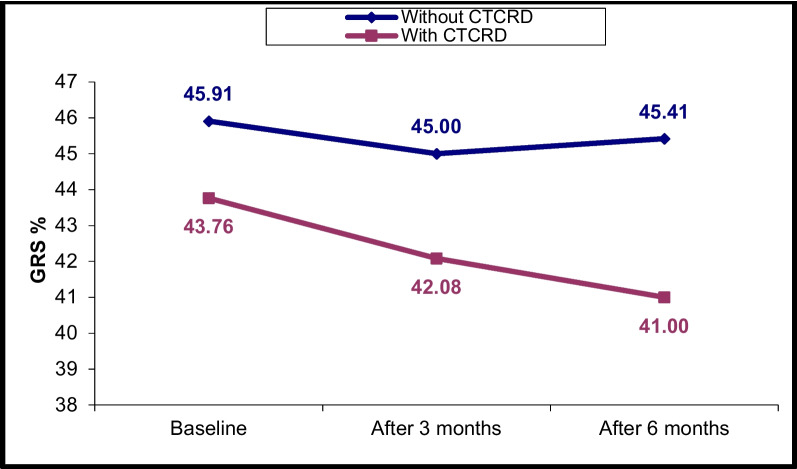
LV GRS% changes in CTRCD and non-CTRCD cases at baseline, after 3 months and after 6 months

**Fig. 6:**
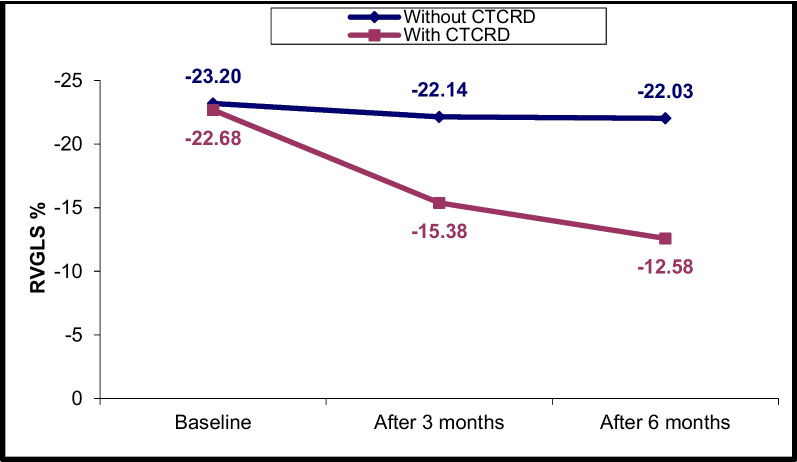
RV GLS% changes in CTRCD and non-CTRCD cases at baseline, after 3 months and after 6 months

**Fig. 7:**
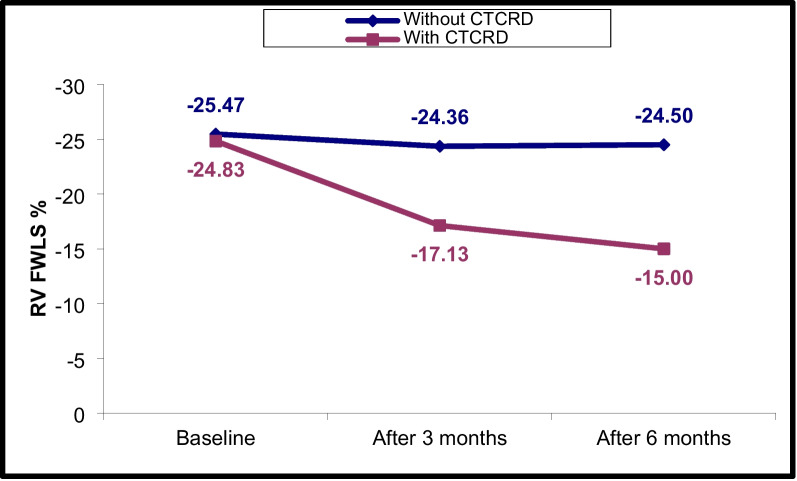
RV FWLS% changes in CTRCD and non-CTRCD cases at baseline, after 3 months and after 6 months

The following figures show the temporal changes in LV and RV mechanics in both CTRCD and non-CTRCD cases (Figs. 1, 2, 3, 4, 5, 6 and 7).

### Predicting a subsequent CTRCD development

On the univariate logistic regression analysis model, baseline LV GLS (< -18%), LV GCS (< -23.9%), RV GLS (< -22.6%), and RV FWLS (< -24%) were predictors of CTRCD occurrence as shown in Table 5. At the 3-month follow-up visit, LV GLS (< -15% with a relative reduction of 12.5% from the baseline) together with RV GLS (< -18%) and RV FWLS (< -19%) were predictors of later CTRCD development. A multivariate regression model showed that the relative reduction of LV GLS of 12.5% at 3 months from baseline was an independent predictor for the development of CTRCD as shown in Table 5.

**Table 5:** Univariate and multivariate logistic regression analysis for factors associated with CTCRD after 6 months

	**Univariate**				**Multivariate**			
	***P*-value**	**Odds ratio (OR)**	**95% C.I. for OR**		***P*-value**	**Odds ratio (OR)**	**95% C.I. for OR**	
			**Lower**	**Upper**			**Lower**	**Upper**
Diabetes Mellitus	**0.000**	6.750	2.445	18.637	–	–	–	–
Trastazumab	**0.006**	4.241	1.500	11.989	–	–	–	–
**Baseline**								
LVESD > 33	**0.040**	2.857	1.050	7.772	–	–	–	–
Diastolic function grade I	0.070	6.815	0.853	54.461	–	–	–	–
EF by M-mode < = 60	**0.010**	3.710	1.360	10.123	–	–	–	–
RV S’ < = 13	0.073	4.098	0.878	19.123	–	–	–	–
GLS % < = -18	**0.001**	9.333	2.546	34.214	–	–	–	–
GCS % < = -23.9	**0.014**	3.385	1.274	8.995	–	–	–	–
RVGLS % < = -22.6	**0.018**	3.172	1.214	8.288	–	–	–	–
RV FWLS % < = -24	**0.040**	2.857	1.050	7.772	–	–	–	–
**After 3 months**								
LVESD > 33	**0.000**	10.217	3.128	33.375	–	–	–	–
EF M mode < = 58	**0.000**	33.889	9.408	122.069	–	–	–	–
EF Simpson’s method < = 55	**0.000**	82.500	18.904	360.046	–	–	–	–
GLS % < = -15	**0.000**	85.250	16.803	432.517	–	–	–	–
RVGLS % < = -18	**0.000**	178.250	21.115	1504.785	–	–	–	–
RV FWLS % < = -19	**0.000**	207.000	24.136	1775.303	–	–	–	–
GLS change < = 12.5%	**0.000**	207.000	24.136	1775.303	**0.000**	198.000	23.046	1701.117
RVGLS change < = 21.74%	**0.000**	198.000	23.046	1701.117	–	–	–	–
RVFWLS change < = 24%	**0.000**	207.000	24.136	1775.303	–	–	–	–

**Fig. 8:**
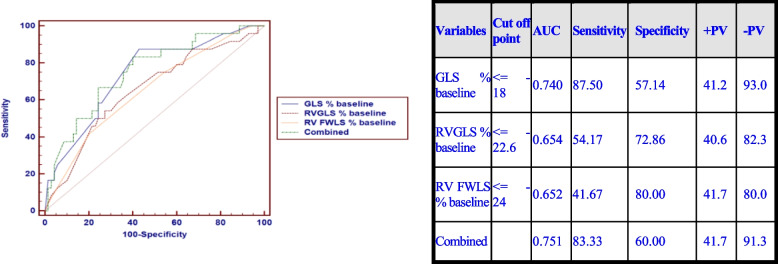
ROC curve for GLS%, RV-GLS% and RV-FWLS% at baseline to detect CTCRD

Based on the Receiver Operating Characteristic (ROC) curve Fig. 8, low values of LV GLS, RV GLS, and RV FWLS at the baseline were predictors of CTRCD development with combining them together was the strongest baseline predictor (AUC = 0.751, sensitivity = 83.33%, specificity = 60%) as shown in Fig. 8.

The integration of cardiac biomarkers such as troponin together with the strain parameters will help us to detect early cardiac abnormalities [7].

**Fig. 9:**
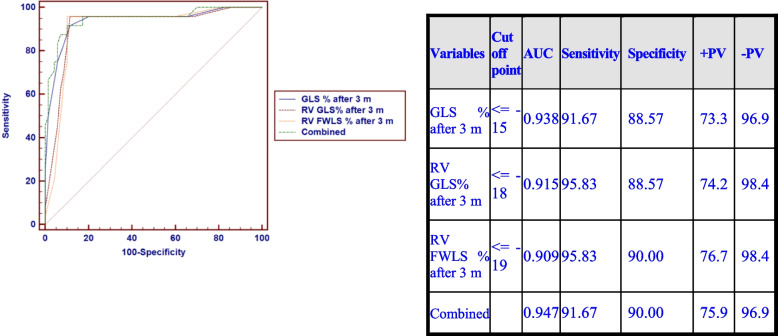
ROC curve for LV GLS, RV GLS, RV FWLS after 3 months to detect CTCRD

After 3 months combining LV GLS, RV GLS, and RV FWLS together was the strongest predictor of the development of CTRCD as shown in Fig. 9 (AUC = 0.947, sensitivity = 91.67%, specificity = 90%).

**Fig. 10:**
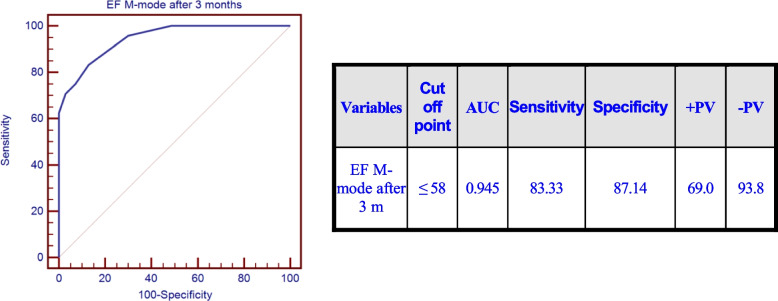
EF by M mode after 3 months to detect CTCRD

This is in comparison with the ROC curve for EF by M mode after 3 months to detect CTRCD as shown in Fig. 10.

## Discussion

Early detection of CTRCD is the main concern of the cardio-oncology team during chemotherapy to be able to start cardio-protective strategies as early as possible. The role of 2D speckle tracking echocardiography in early detection of CTRCD was previously tested in many studies. Alterations in strain parameters of 2D STE have been shown to precede a decrease in EF [15]. In this present study, we assessed the deformation mechanics in both LV and RV throughout a 6-month chemotherapy regimen. First of all, in our study changes in RV mechanics were more frequent than LV at the end of the follow up period. RV dysfunction occurred in 37 patients representing 39.4% of the study population with almost half of them occurring as early as the first follow-up visit after 3 months (T1). On the contrary, LV dysfunction was less prevalent and more delayed occurring in 25.5% of the cases with 45% of them at (T1). These results were consistent with the results shown by Zhao et al., 2020 in which the drop in RV function preceded LVEF decrease [16]. The underlying suggested reason was the fact that the RV wall is thinner, having less reserve for compensation compared to the LV, and thus more prone to cardiotoxicity. This is also confirmed by other imaging modalities, as evidenced by a study from Ylanen et al., 2013 which evaluated the late effects of Anthracycline-induced cardiotoxicity in childhood cancer survivors, using cardiac MRI. This study showed a 27% prevalence of abnormal RV function and only an 18% prevalence of abnormal LV function [17]. On the other side one study from Keramida et al., 2019 showed that LV GLS was significantly reduced after 3 months of chemotherapy earlier than RV GLS [18]. Although there was no postulated hypothesis justifying this earlier LV affection, it can be assumed to differences in the inclusion criteria of the study population in this study from our study. At Keramida et al., 2019, 54.5% of the study population received previous radiotherapy treatment, unlike our study in which no patient received previous radiotherapy treatment. This controversy necessitates more studying of the change in RV mechanics during chemotherapy & comparing it to LV changes although the hypothesis of the thinner RV wall seems reasonable, but more evidence is still required.

Regarding the changes in LV strain parameters, our study showed that LV GLS is the most sensitive strain parameter to predict later CTRCD. At baseline, people with lower LV GLS values –even may be still in the normal range- were associated with a higher risk for developing later CTRCD. In this study, a Baseline LV GLS value less than -18% was associated with an increased possibility of CTRCD development than higher values (AUC = 0.740, sensitivity = 87.5%, specificity = 57.14%). The suggested explanation was that cancer itself is considered as an inflammatory process releasing various reactive oxidative species and combined with neuro-hormonal changes. This has a significant effect on myocardium which is usually aggravated by chemotherapy [18], however, the possibility of the patients with lower baseline strain values may have an underlying cardiac disease should also be considered.

At the 3-month follow-up visit (T1), changes in LV GLS value were stronger predictors than LV GCS and GRS as evidenced by univariate and multivariate regression analysis models (using LV GLS cut-off value of -15% or a decrease from the baseline value by 12.5%). This result was consistent with the previous studies and with ASE/EACVI consensus guidelines which recommended measurement of GLS for follow-up of cancer patients receiving chemotherapy [8]. The postulated hypothesis behind this is that cardiotoxicity results from regional deformational changes of the myocardium which also explains why LV strain changes precede LV EF changes because some segments are affected usually earlier than others which on the contrary plays a compensatory role and thus LV EF remains stable. The long-axis function of the left ventricle is governed by subendocardial, longitudinally-arranged myocardial fibers, which are more susceptible to early hypoxia and myocardial injury [4]. That’s why in the case of early myocardial injury, longitudinal strain is usually affected first while circumferential strain may compensate for left ventricular function. With the progression of the disease, circumferential strain will be affected which might indicate transmural involvement [19].

In this study, our primary endpoint was trying to detect the earliest strain parameter that precedes EF changes and thus can predict CTRCD later on. In our study and based on the ROC curve, combining LV GLS with RV GLS and RVFWLS was the best predictor of subsequent CTRCD. Although there is a paucity of data in the literature studying the role of RV strain parameters in predicting LV EF drop, our study results are consistent with the results of Arciniegas Calle et al. published in 2018 [20]. At Arciniegas Calle et al., 2018, it was concluded that Combining both RV GLS at T1 and LV GLS was the strongest predictor of cardiotoxicity (area under the curve[AUC], 0.91; sensitivity, 100%; specificity, 73%; P < 0.001). LV GLS at T1 (AUC, 0.85; cutoff, − 14.06; sensitivity, 91%; specificity, 83%; *P* = 0.003) was also a strong indicator of subsequent cardiotoxicity. This can be explained because it is known that usually, longitudinal deformational changes are always the earliest to happen and thus measuring longitudinal RV strain would increase the ability to predict the future drop in EF because more muscle fibers are studied and the wall of the RV is even thinner than LV and thus it would be more affected.

We aimed to highlight the importance of studying the changes in RV mechanics during the period of chemotherapy as combining some of these data with LV strain variables gives us the best chance to predict CTRCD and detect it as early as possible, so we can modify the chemotherapy regimen and start cardio-protective measures early and to detect patients based on their baseline data who are at risk of later development of CTRCD.

### Study limitations

Our study limitations were that it was a single-center study and the duration of follow-up was 6 months only. Since the follow up period is shorter than the trastuzumab full cycle (usually 12 months), trastuzumab induced cardiotoxicity couldn’t have been conclusively evaluated by this study. Also, we didn’t study whether these early cardiotoxic changes are reversible in a longer follow-up duration or not.

## Conclusion

Anthracycline ± Trastuzumab treatment leads to changes in LV & RV mechanics with more prevalent deterioration in RV values. The presence of lower LV & RV strain values at the base line together with reduction of these values after chemotherapy treatment can predict the later development of CTRCD. Combining LV GLS with RV GLS & FWLS values at (T1) is the strongest predictor of subsequent CTRCD.

## Data Availability

The data of the studied patients are available with the authors upon request.

## References

[1] Bray F, Ferlay J, Soerjomataram I, Siegel RL, Torre LA, Jemal A (2018). Global cancer statistics 2018: GLOBOCAN estimates of incidence and mortality worldwide for 36 cancers in 185 countries. CA Cancer J Clin.

[2] Chen W, Jiao Z, Li W, Han R (2022). Two-dimensional speckle tracking echocardiography, a powerful method for the evaluation of anthracyclines induced left ventricular insufficiency. Medicine.

[3] ElZarrad MK, Mukhopadhyay P, Mohan N, Hao E, Dokmanovic M, Hirsch DS (2013). Trastuzumab alters the expression of genes essential for cardiac function and induces ultrastructural changes in cardiomyocytes in mice. PLoS ONE.

[4] Cascino GJ, Voss WB, Canaani J, Furiasse N, Rademaker A, Ky B (2019). Two-dimensional speckle-tracking strain detects subclinical cardiotoxicity in older patients treated for acute myeloid leukemia. Echocardiography.

[5] Santoro C, Arpino G, Esposito R, Lembo M, Paciolla I, Cardalesi C (2017). 2D and 3D strain for detection of subclinical anthracycline cardiotoxicity in breast cancer patients: a balance with feasibility. Eur Heart J Cardiovasc Imaging.

[6] Cardinale D, Colombo A, Lamantia G, Colombo N, Civelli M, De Giacomi G (2010). Anthracycline-induced cardiomyopathy: clinical relevance and response to pharmacologic therapy. J Am Coll Cardiol.

[7] Sawaya H, Sebag IA, Plana JC, Januzzi JL, Ky B, Tan TC (2012). Assessment of echocardiography and biomarkers for the extended prediction of cardiotoxicity in patients treated with anthracyclines, taxanes, and trastuzumab. Circulation.

[8] Plana JC, Galderisi M, Barac A, Ewer MS, Ky B, Scherrer-Crosbie M (2014). Expert consensus for multimodality imaging evaluation of adult patients during and after cancer therapy: a report from the American Society of Echocardiography and the European Association of Cardiovascular Imaging. Eur Heart J Cardiovasc Imaging.

[9] Williams B, Mancia G, Spiering W, AgabitiRosei E, Azizi M, Burnier M (2018). 2018 ESC/ESH Guidelines for the management of arterial hypertension: The Task Force for the management of arterial hypertension of the European Society of Cardiology (ESC) and the European Society of Hypertension (ESH). Eur Heart J.

[10] American Diabetes Association (2010). Diagnosis and classification of diabetes mellitus. Diabetes Care.

[11] Levey AS, Eckardt KU, Tsukamoto Y, Levin A, Coresh J, Rossert J (2005). Definition and classification of chronic kidney disease: a position statement from Kidney Disease: Improving Global Outcomes (KDIGO). Kidney Int.

[12] Lyon AR, Lopez-Fernandez T, Couch LS, Asteggiano R, Aznar MC, Bergler-Klein J (2022). 2022 ESC Guidelines on cardio-oncology developed in collaboration with the European Hematology Association (EHA), the European Society for Therapeutic Radiology and Oncology (ESTRO) and the International Cardio-Oncology Society (IC-OS) Developed by the task force on cardio-oncology of the European Society of Cardiology (ESC). Eur Heart J Cardiovasc Imaging.

[13] Lang RM, Badano LP, Mor-Avi V, Afilalo J, Armstrong A, Ernande L (2015). Recommendations for cardiac chamber quantification by echocardiography in adults: an update from the American Society of Echocardiography and the European Association of Cardiovascular Imaging. Eur Heart J Cardiovasc Imaging.

[14] Marwick TH (2006). Measurement of strain and strain rate by echocardiography: ready for prime time?. J Am Coll Cardiol.

[15] Poterucha JT, Kutty S, Lindquist RK, Li L, Eidem BW (2012). Changes in left ventricular longitudinal strain with anthracycline chemotherapy in adolescents precede subsequent decreased left ventricular ejection fraction. J Am Soc Echocardiogr.

[16] Zhao R, Shu F, Zhang C, Song F, Xu Y, Guo Y (2020). Early detection and prediction of anthracycline-induced right ventricular cardiotoxicity by 3-dimensional echocardiography. Cardio Oncology.

[17] Ylänen K, Poutanen T, Savikurki-Heikkilä P, Rinta-Kiikka I, Eerola A, Vettenranta K (2013). Cardiac magnetic resonance imaging in the evaluation of the late effects of anthracyclines among long-term survivors of childhood cancer. J Am Coll Cardiol.

[18] Keramida K, Farmakis D, Bingcang J, Sulemane S, Sutherland S, Bingcang RA (2019). Longitudinal changes of right ventricular deformation mechanics during trastuzumab therapy in breast cancer patients. Eur J Heart Fail.

[19] Bansal M, Sengupta PP (2013). Longitudinal and circumferential strain in patients with regional LV dysfunction. Curr Cardiol Rep.

[20] Arciniegas Calle MC, Sandhu NP, Xia H, Cha SS, Pellikka PA, Ye Z (2018). Two-dimensional speckle tracking echocardiography predicts early subclinical cardiotoxicity associated with anthracycline-trastuzumab chemotherapy in patients with breast cancer. BMC Cancer.

